# Snails from heavy-metal polluted environments have reduced sensitivity to carbon dioxide-induced acidity

**DOI:** 10.1186/s40064-015-1073-9

**Published:** 2015-06-17

**Authors:** Hugh Lefcort, David A Cleary, Aaron M Marble, Morgan V Phillips, Timothy J Stoddard, Lara M Tuthill, James R Winslow

**Affiliations:** Biology Department, Gonzaga University, 502 E. Boone Avenue, Spokane, WA 99258 USA; Chemistry Department, Gonzaga University, 502 E. Boone Avenue, Spokane, WA 99258 USA

**Keywords:** Snails, Carbon dioxide, Climate change, Heavy metals, Behavior

## Abstract

Anthropogenic atmospheric CO_2_ reacts with water to form carbonic acid (H_2_CO_3_) which increases water acidity. While marine acidification has received recent consideration, less attention has been paid to the effects of atmospheric carbon dioxide on freshwater systems—systems that often have low buffering potential. Since many aquatic systems are already impacted by pollutants such as heavy metals, we wondered about the added effect of rising atmospheric CO_2_ on freshwater organisms. We studied aquatic pulmonate snails (*Physella columbiana*) from both a heavy-metal polluted watershed and snails from a reference watershed that has not experienced mining pollution. We used gaseous CO_2_ to increase water acidity and we then measured changes in antipredatory behavior and also survival. We predicted a simple negative additive effect of low pH. We hypothesized that snails from metal-polluted environments would be physiologically stressed and impaired due to defense responses against heavy metals. Instead, snails from populations that acclimated or evolved in the presence of heavy metal mining pollution were more robust to acidic conditions than were snails from reference habitats. Snails from mining polluted sites seemed to be preadapted to a low pH environment. Their short-term survival in acidic conditions was better than snails from reference sites that lacked metal pollution. In fact, the 48 h survival of snails from polluted sites was so high that it did not significantly differ from the 24 h survival of snails from control sites. This suggests that the response of organisms to a world with rising anthropogenic carbon dioxide levels may be complex and difficult to predict. Snails had a weaker behavioral response to stressful stimuli if kept for 1 month at a pH that differed from their lake of origin. We found that snails raised at a pH of 5.5 had a weaker response (less of a decrease in activity) to concentrated heavy metals than did snails raised at their natal pH of 6.5. Furthermore, snails raised a pH of 5.5, 6.0, and 7.0 all had a weaker antipredatory response to an extract of crushed snail cells than did the pH 6.5 treatment snails.

## Background

It has long been recognized that atmospheric pollutants and contact with mining wastes could impact aquatic habitats (Literary Digest 1926; Berl 1934; Ellis 1940). During the 1970’s and 1980’s it was thought that the main cause of atmospheric pollution was nitric and sulfuric acid formation due to the burning of fossils fuels (Schindler et al. 1985). By the first part of the twenty-first Century it began to be apparent that massive anthropogenic atmospheric carbon dioxide inputs could also affect the oceans by increasing water acidity (Caldeira and Wickett 2003). Since then a large body of literature has developed that document these effects on marine biota (extensively reviewed in Feely et al. 2012). What have received less attention are the effects of atmospheric carbon dioxide on aquatic systems.

Atmospheric CO_2_ levels have risen from 280 ppm in the year 1800 to around 400 ppm today which are higher than levels in the last 800,000 years (IPCC 2007). Although nutrients can cause the growth of autotrophs which can lower CO_2_ levels (Hickey and Banas 2003), and although atmospheric CO_2_ levels have been much higher several times over the last 300 million years, they have not risen at such a rapid rate. A slow rise allows natural events like the weathering of rocks to buffer the change but today’s brisk rise can overtake these processes (Archer et al. 1997). This rise may affect gastropods which form the basis of many aquatic food chains.

Carbon dioxide increases water acidity by:

$${\text{CO}}_{{2 \, ({\text{g}})}} \rightleftarrows {\text{CO}}_{2 \, (aq)} + {\text{ H}}_{2} {\text{O}}_{(l)} \rightleftarrows {\text{H}}_{2} {\text{CO}}_{3\;(aq)} \rightleftarrows {\text{H}}_{(aq)}^{ + } + {\text{ HCO}}_{3\;(aq)}^{ - }$$

and

$${\text{H}}_{(aq)}^{ + } + {\text{CO}}_{3\;(aq)}^{2 - } \rightleftarrows {\text{HCO}}_{3\;(aq)}^{ - }$$

Atmospheric CO_2_ reacts with water to form carbonic acid (H_2_CO_3_). Carbonic acid then dissociates into a hydrogen ion (H^+^), and bicarbonate, HCO_3_^−^. Some of the H^+^ released by the carbonic acid reacts with carbonate (CO_3_^2−^) that is already in the water to form more bicarbonate. This loss of carbonate is serious because many gastropods use it to build shells of calcium carbonate (CaCO_3_). The rise in acidity reduces the saturation state of aragonite which is a biomineral of calcium carbonate (Caldeira and Wickett 2003; Orr 2005; Feely et al. 2012). Once the saturation state drops below a certain level gastropods shells are thermodynamically favored to corrode. The animals can overcome this loss of shell thickness but only by using an energetically expensive process to lay material onto the inner surface of the shell (Fabry et al. 2008). Contrarily, a rise in water temperature makes CO_2_ less soluble in water (Feely et al. 2012). In addition, dissolution of shells is also affected by species specific protective organic coatings on shells, trace element components, and the microstructure of the shell (Feely et al. 2009).

Many marine organism exhibit impaired growth and development under high pCO_2_ levels. For example, Northern Abalone (*Haliotis kamtschatkana*) is a marine gastropod that experiences deformed larval morphology when raised under an atmosphere of 800 ppm pCO_2_ (Crim et al. 2011). Several studies have found impaired growth of mussels (*Mytilus*) under high pCO_2_ conditions (Gaylord et al. 2011; Gazeau et al. 2011) but two meta-analyses of molluscs (Hendriks et al. 2010; Kroeker et al. 2010) did not find a pCO_2_ effect on growth. Furthermore, in some oysters the effect of elevated water acidity on growth and survival is ameliorated in the second generation (Feely et al. 2009).

Marine organism under high pCO_2_ levels also exhibit behavioral alterations. Acidic water makes hermit crabs more risk adverse (de la Haye et al. 2011) and impairs the ability of hermit crabs (de la Haye et al. 2012) and reef fishes (Dixson et al. 2010, 2012) to detect chemical alarm cues. Conch snails exhibit a reduced ability to “leap” backwards when confronted by cone shell predators (Watson et al. 2014).

The effects of atmospheric CO_2_ on freshwater are less well known since most studies of freshwater acidity concerned nitric acid and sulfuric acid caused acid rain (Økland and Økland 1986; Mason 1992; Korsman 1999; Lacoul et al. 2011). In these studies some pulmonate snails exhibit sensitivities below a pH of only 6.25 (Holcombe et al. 1984) but others have found an Amnicolid freshwater snail that was robust to short-term exposure of nitric acid with a pH of 4.0 (Servos and Mackie 1986).

In addition to direct physiological effects (Økland and Økland 1979) acidic water can impact predator/prey interactions (Lippert et al. 2007) in a wide range of taxa. Acid water impairs the ability of juvenile freshwater salmon (Leduc et al. 2006, 2009) to detect and avoid fish predators, and rainbow trout (Brown et al. 2012) fail to detect chemical alarm cues. In addition, acidic water negatively impacts the ability of beetles to use temperature changes to moderate diving behavior (Calosi et al. 2007) and pulmonate snails are more common in alkaline streams (Dillon and Benfield 1982). High alkalinity can have a similar effect in that at pH 7.5 *Physa acuta* snails respond to fish cues by moving into safer habitats, but avoidance becomes impaired at a pH of 9.4 (Turner and Chislock 2010).

### Metal pollution

Anthropogenic influences rarely occur alone; along with CO_2_ rises, organisms also are affected by co-occurring pollutants such as heavy metals. Increasingly, researchers are beginning to look at the synergistic, antagonistic, and additive effects of multiple stressors (Heugens et al. 2001, Coors and De Meester 2008) especially CO_2_ induced climate effects (Noyes et al. 2009; Hooper et al. 2013; Moe et al. 2013). Acidity may alter the solubility of metals and increase their toxicity when in a dissolved state since metals tend to be more harmful in soft water (Hunter 1980; Feely et al. 2012). In waters with low alkalinity, acidification increased the toxicity of copper to larval fathead minnow (Welsh et al. 1993). Yet the reverse is also observed. Dissolved zinc is more lethal to rainbow trout when the water has a *higher* pH and lower hardness levels (Bradley and Sprague 1985). Furthermore, in a test of an amphipod, the toxicity of cadmium and zinc was greatest at pH 8.3 and least at pH 6.3 while the toxicity of lead was greatest at pH 6.3 and least at pH 8.3 (Schubauer-Berigan et al. 1993).

One area where the combined impacts of CO_2_-induced acidity and metal pollution may be observed is in the Silver Valley of northern Idaho, USA. For over 130 years pollution from mine tailings have been released into the environment (Neufeld 1987; Farag et al. 1998; Sprenke et al. 2000). Metals such as lead, zinc, cadmium, and arsenic are patchily distributed in downstream lakes (Ellis 1940; Rabe and Bauer 1977; Ridolfi Engineering 1993). The lakes have been contaminated for over 125 years and hence at least 125 snail generations (Hunter 1975; Lefcort et al. 2010) have undergone selective pressures due to heavy metals.

Our lab examines how aquatic snails (*Physella columbiana*) avoid patchy distributions of heavy metal pollution while also balancing the need to avoid predation (Lefcort et al. 1999, 2002, 2008). We study snails from both a heavy metal polluted watershed and snails from a reference watershed that has not experienced mining. Snails are a good model for study because they are a major component of the metal-impacted food chains. Snails have contact with bottom sediments and they are a preferred food for centrarchids and salmonids fishes (Ellis 1940).

These snails alter their behavior when exposed to kairomones released from crushed snail cells which are an indicator of predation (von Frisch 1938; Atema and Stenzler 1977; Dickey and McCarthy 2007). Upon detection the snails reduce their movements and pull into their shells (Lefcort et al. 2000). Metals stress the snails and impair this response (Lefcort et al. 2000).

Since anthropogenic CO_2_ is rising we wanted to revisit some of the work done in the 1970’s and 1980’s concerning acid rain while looking at a new stressor—carbonic acid. Specifically, does CO_2_-induced water acidity have different effects than nitric acid or sulfuric acid-induced water acidity? Also how does a new toxicant, CO_2_, affect the survivability and antipredatory behavior of organisms that exist in an ecosystem already polluted by heavy metal mining waste? Are the effects of metals and acidity additive or does one ameliorate the other? We predicted that:

1. *P. columbiana* snails from heavy metal lakes would exhibit lower survivability at low pH levels (4.5 and 5.0) than snails from reference lakes.
2. When raised in CO_2_-induced low pH aquatic environments, the snails would exhibit reduced anti-predatory and metal avoidance behaviors.

## Methods

### Experiment 1: LD_50_

We first examined the lethality of CO_2_-induced acidity to two groups of snails; snails from reference sites and snails from sites of known heavy metal pollution. Water acidity and levels of metals in snails from these sites are shown in Table 1.

**Table 1:** Metal levels (mg/kg) of dried unshelled snails used in tests and pH of lakes (X ± SD)

	Cadmium	Lead	Zinc	pH
Reference lakes				
Areush	*	*	4.48	7.3 (0.4)
Badger	*	0.53	5.78	7.0 (0.4)
Bayit	*	*	*	7.1 (0.3)
Deer	*	0.63	14.10	6.7 (0.4)
Hauser	*	*	8.10	6.9 (0.2)
Hick	*	*	4.93	7.2 (0.2)
Mecca	*	*	2.33	7.0 (0.1)
Worm	*	1.30	6.29	6.8 (0.4)
Williams	*	*	5.35	7.0 (0.3)
Metal polluted lakes				
Anderson	*	5.59	11.20	7.3 (0.5)
Bull Run	*	7.15	24.80	7.0 (0.4)
Farm	*	*	14.15	6.7 (0.3)
Goose	*	*	14.35	7.1 (0.2)
Killarney	*	0.71	15.86	7.1 (0.3)
Mile 109	*	*	13.81	7.2 (0.2)
Mile 109.5	*	0.79	14.66	7.2 (0.4)
Porter	*	1.29	16.65	6.9 (0.4)
Rose	*	7.37	34.56	6.9 (0.3)
Thompson		5.53	10.60	6.9 (0.5)

* Below detection limits Cd 0.35 mg/kg, Pb 0.51 mg/kg, Zn 0.44 mg/kg.

We measured the lethal dose of acidity that killed 50% of the population. During June of 2014 we collected *P. columbiana* snails from nine reference lakes and 10 heavy metal polluted lakes. All lakes were along the NE Washington State/NW Idaho State border and were of similar ecological parameters (Lefcort et al. 2000). Thirty snails from each lake were collected and divided into individual 200 mL cups kept at two different levels of acidity by bubbling in CO_2_: pH 4.5 and pH 5.0, for a total of 570 cups. A pH of 4.5 is equivalent to an atmospheric CO_2_ level of 100,000 ppm while a pH of 5.0 is equivalent to 10,000 ppm. Preliminary experiments had shown that all animals died within 12 h at pH 4.0 and all animals survived for 72 h at pH 6.5. The pH of the cups was kept in a range of ±0.3 pH by changing the water every 3 h. No food was provided. The snails were monitored for 48 h, and were marked dead or alive at each hour. An Accumet AB200 pH/Conductivity meter by Fisher Scientific was used to measure pH.

#### Metal analysis

Snail tissues were first dissolved in trace metal grade nitric acid (Sigma-Aldrich Co.) using a CEM brand microwave dissolution (model MDS-2100) system. The solubilized material from snails was then diluted with distilled water and analyzed by Inductively Coupled Plasma Optical Emission Spectrometer and Graphite Furnace Atomic Absorption. All preparations and tests were performed by Anatek Labs Spokane, WA, USA.

### Experiment 2: avoidance and activity

Next we explored the effects of acidic rearing regimes on snail behavior. We raised the snails at four different levels of acidity and then we measured their ability to avoid heavy metals and an index of predation.

#### Rearing snails

We conducted experiments on snails from Bayit Pond, Spokane County, WA, USA). Bayit Pond is a reference pond (128 m^2^) that is free of heavy metals and has a mean (±SE) pH of 6.87 (0.24). It was constructed in 2003 and stocked with snails from Coeur d’Alene Basin lakes in 2008.

In May of 2013 two hundred snails were brought into our lab at Gonzaga University and housed in a 40-L aquaria filled with artificial pond water (deionized water, CaCl_2_, K_2_HPO_4_, and MgSO_4_ that resulted in water properties of calcium 4.84 mg/L, chloride 3.52 mg/L, and conductivity of 48.6 µmhos/cm). All were fed lettuce and none were reared with heavy metals. Water was changed twice weekly. Fifty animals were raised for 1 month at either pH 7.0, 6.5. 6.0 or 5.5. In preliminary experiments we also used a pH 5.0 treatment but few animals survived.

#### Controlling pH

Filtered beverage grade pure carbon dioxide (OXARC, Spokane, WA, USA) was filtered and then bubbled through an air stone to reduce the pH of certain treatments (the pH 7.0 treatment was bubbled with atmospheric air). The CO_2_ was kept at a constant pressure of approximately 100 kPa and added to the tubs for differing amounts of time, twice a day until the desired pH was attained. This kept the pH in a range of ±0.3 pH units. A similar diurnal variation in pH also naturally occurs at our field sites (Lefcort unpubl.).

It was difficult to achieve a pH of 7.0 since un-buffered water exposed to ambient laboratory conditions interacted with high anthropogenic levels of atmospheric CO_2_ and equilibrated at ~pH 6.5. Therefore we buffered each tub using Tetra^®^ pH 7.0 tablets which is composed primarily of sodium bicarbonate. In ponds, the normal dissolution of naturally occurring buffers, such as limestone, results in natural bicarbonate which has the same effect. The artificial pond water solution plus the Tetra tablet resulted in water properties of calcium 5.74 mg/L, chloride 30.7 mg/L, sodium 76.6 mg/L, and conductivity of 232.0 µmhos/cm.

#### Preparation of metal solutions

We prepared heavy metal soil stock solution by stirring one liter of vigorously rinsed soil from a river bank within the Bunker Hill Superfund site in northern Idaho State (Lefcort et al. 1999), into a container of 40 L of deionized water. After letting the solution settle for 48 h we poured off the supernant and filtered it. After dilutions this resulted in a concentration of 2.1 µg/L of cadmium, 6,500.0 µg/L of zinc, 105.6 µg/L of lead, and minor amounts of other metals (Anatek Labs, Spokane, WA, USA using inductively coupled plasma mass spectrometry and graphite furnace atomic absorption). Dilutions were then made with artificial pond water. For all experiments pond water was freshly prepared on the day of testing.

#### Preparation of crushed-snail extract

We produced snail extract (an inducer of a fright response, Lefcort et al. 2000, 2013) by first slowly cooling a snail (0.4–0.6 g) to 3°C in a dish of pond water. The animal was then macerated with 50 mL of DI water. One drop of this extract was used in 2 L of test solution. No anesthesia other than cooling was used to avoid introducing novel chemical cues (approved by Gonzaga Institutional Animal Care and Use Committee).

#### Testing snails

**Figure 1:**
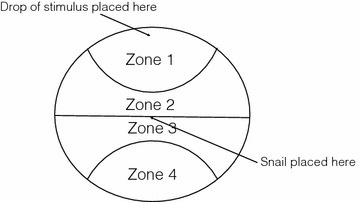
Avoidance dish used in Experiment 2.

Twenty-five snails from each of the four pH rearing levels were tested using a behavioral assay previously used in our lab (Lefcort et al. 2013). A 15 cm diameter circular glass dish (Figure 1) was divided into four equal sized zones. The dish was covered in a thin layer of pre-washed sand and artificial pond water. A snail was placed in the middle of the dish, on the midline between zones 2 and 3. At each minute, for 20 min, the snail’s zone was recorded to determine if the snail moved away from the stimulus (scored as *avoidance*). Each snail was used for three tests (in three separate dishes) in the following order: control—with no added stimulus, extract—using a drop of snail extract in zone 1, and heavy metal avoidance—using a drop of heavy metal solution in zone 1.

Snails were rested between treatments one and two, and between two and three for 60 min in a dish of pond water. Individual snails were only tested in a single set of experiments. This was done to prevent any effects of learning. Previous experiments (Lefcort et al. 2013) showed that repeated trials of the same treatment did not alter individual snail responses, i.e., there was no time effect, and the order of stimuli was not significant as long as 60 min elapsed between trials. The dishes were washed with hot water and wiped dry with paper towels between animal replicates and the sand was changed.

#### Statistical tests

Probit analysis (Finney 1947) was used to determine calculated LD_50_ values in Experiment 1 followed by Student’s t tests and one-way analysis of variance followed by Newman–Keuls multiple comparisons. Kruskal–Wallis one-way analysis of variance by ranks was used in Experiment 2 after nonparametric adjusted rank transform tests (Leys and Schumann 2010) indicated no significant interaction terms. Alpha was set to 0.05.

## Results

### Experiment 1: LD_50_

**Figure 2:**
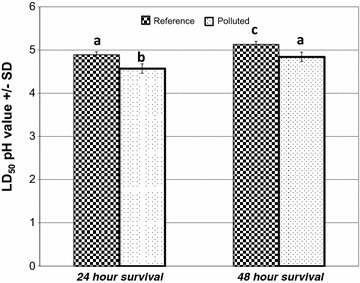
Survival of snails in acidic water. Snails were from nine reference and ten heavy metal polluted lakes. Survivability was measured each hour for 48 h; 24 and 48 h values are shown for illustration.

Survivorship values (based on hydrogen ion levels) are displayed in Figure 2. Snails from heavy-metal polluted ponds had higher survival than snails from reference ponds. This occurred at both 24 h (t = 2.43, df = 17, P = 0.032) and 48 h (t = 3.47, df = 17, P = 0.003). The 48 h survival of snails from polluted sites was so high that it not significantly differ from the 24 h survival of snails from control sites (one-way ANOVA F_3,36_ = 8.85, P < 0.001, Newman-Keuls multiple comparisons P < 0.05). Acidity values of metal-polluted and reference lakes did not differ (t = 1.45, df = 17, P = 0.608). For unknown reasons one reference lake, Deer Lake, had high zinc levels. We included this lake in the above analyses but the results were similar when it was excluded.

### Experiment 2A: movement, activity in relation to stimulus

**Figure 3:**
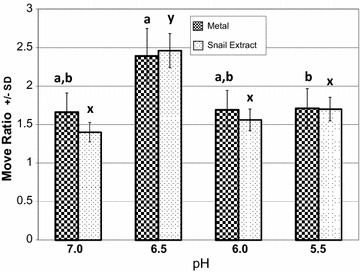
Movement and rearing pH. The number of times each animal (X ± SD) crossed between zones was continuously recorded for 20 min. Y values were calculated by dividing control stimulus (plain pond water) moves by the experimental stimulus (extract or metal) moves. Hence, Y axis values above 1 indicate that the animal moved less during experimental treatments than under control conditions. *Letters* are Tukey multiple comparisons. *Identical letters* indicate no significant difference between values.

The response of the animals to metal solutions (Kruskal–Wallis H = 9.62, df = 3, p = 0.023) and crushed-snail extract (Kruskal–Wallis H = 20.28, df = 3, p < 0.001, Figure 3) were both impaired if the snails were raised at a pH that was closer to their natal acidity of roughly 6.5 (Figure 3). Animals had a stronger (less movement) response to both metals and snail extract if raised at a pH of 6.5. Snails raised at higher pH (7.0) or lower pH (6.0 and 5.5) significantly decreased their movements (when compared to control water) but not as much as the snails raised at pH 6.5.

### Experiment 2B: avoidance, location due to stimulus

**Figure 4:**
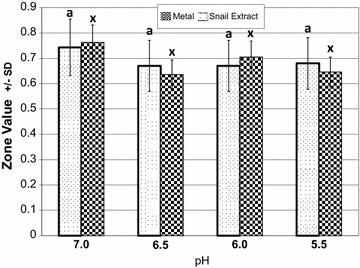
Avoidance and rearing pH. Y axis (zone location) is determined by assigning a linear point value for each of eight zones. Zones close to the stimulus drop had low values and zones further away had higher values. Zones locations were recorded each minute for 20 min. These 20 values were added to derive one number for control stimulus (pond water), one for extract, and one for metal stimulus. Y values were calculated by dividing control stimulus value by the experimental stimulus (extract or metal) value. Hence, Y axis values below 1 indicate that the experimental animals moved further away from the stimulus more than they had moved under control stimulus*. Letters* are Tukey multiple comparisons. *Identical letters* indicate no significant difference between values.

Water acidity did not alter the locations of the animals over the 20 min test period when exposed to metal stimulus (Kruskal–Wallis H = 4.28, df = 3, p = 0.234, Figure 4) nor snail extract (Kruskal–Wallis H = 5.05, df = 3, p = 0.169, Figure 4), i.e., rearing acidity did not increase or decrease the snails’ natural avoidance of metals and extract.

## Discussion

We found that snails that evolved in the presence of heavy metal mining pollution were more robust to acidic conditions than were snails that had not evolved in the presence of heavy metals. This suggests that the response of organisms to a world with rising anthropogenic carbon dioxide levels may be more complex than once believed.

We predicted a simple negative additive effect of low pH. We hypothesized that snails from metal-polluted environments would be physiologically stressed and impaired due to defense responses against heavy metals. What we found was that these snails seem to be preadapted to a low pH environment. Their short-term survival in acidic conditions (Experiment 1) was better than snails from reference sites that lacked metal pollution. In fact, the 48 h survival of snails from polluted sites was so high that it did not significantly differ from the 24 h survival of snails from control sites. The mechanism behind this result is unknown but it may be due to altered epithelium membrane permeability (Sullivan and Cheng 1975) or altered mucus secretion. Pedal mucus secretion by closely related *Lymnaea stagnalis* snails reduce the effects of aluminum exposure (Jugdaohsingh et al. 1998; Balance et al. 2001) but it is unknown if this would be protective in high acidity environments. Our reference site snails seem similar to a closely related Physid snail which exhibited sensitivities at pH values below 6.25 (Holcombe et al. 1984).

This is not to say that rising global atmospheric CO_2_ levels may not have an overall negative effect on snails. We also observed that snails had a weaker response to stressful stimuli if kept for 1 month at a pH that differed from their natal lake (Experiment 2). These snails were derived from a population inhabiting a heavy metal rich environment but they have spent the last five generations in a heavy metal-free pond whose pH annually ranges from 6.5 to 7.1.

Molluscs may be quite susceptible to changes in water quality. Freshwater mussels experience oxidative damage when exposed to many of the component of urban runoff (Machado et al. 2014). In particular a drop in pH may pose particular challenges to gastropods and hence our results are similar to those of the 1970’s and 1980’s that examined the effects of acid rain (Økland and Økland 1979; Servos and Mackie 1986). For example marine abalone exposed to acidic waters have impaired feeding abilities as larvae (Vargas et al. 2013), and adults are less able to self-right themselves after dislodgement (Manríquez et al. 2013). Mussels have reduced phagocytic ability at higher acidity levels (Bibby et al. 2008).

Snails normally reduce their activity when exposed to an extract of crushed snail cells or when exposed to concentrated heavy metals (Lefcort et al. 2000, 2013). However we found that snails raised at a pH of 5.5 had a weaker response (less of a decrease in activity) to concentrated heavy metals than did snails raised at a pH of 6.5. Furthermore, snails raised at a pH of 5.5, 6.0, and 7.0 all had a weaker response to an extract of crushed snail cells than did the pH 6.5 treatment snails. In addition to a reduction of activity, snails also normally move away from a drop of heavy metal rich water or an extract of crushed snail cells (Lefcort et al. 2013). In this study we found that this avoidance response was not significantly affected by a 1 month exposure to an altered level of water acidity. Our results are similar to a study of mangrove-inhabiting periwinkles that moved away from acidified waters (pH 6.2–7.0, Amaral et al. 2014). One intertidal periwinkle normally produces thicker shells in the presence of crab predatory cues but this response is disrupted at low seawater pH (Bibby et al. 2007).

Yet the net effect of higher acidity levels is unclear. Acidic runoff in estuaries revealed no effects on crabs and only weak effects on bivalves and gastropods (Amaral et al. 2011). Indirect effects may also allow certain taxa to benefit from the impairment of competing taxa (Barry et al. 2013). Additionally, the effects of acidification on antipredatory responses have been found to be quite species specific among coral reef fishes (Ferrari et al. 2011). Even organisms as taxonomically distant from gastropods as freshwater diatoms, experience a nonadditive effect of heavy metals and acidity (Luís et al. 2014). These diatoms live in biofilms near the output of hardrock mines. Populations that have adapted to low pH conditions are less affected by copper and zinc effluents (Luís et al. 2014).

Given the behavioral effects of rising CO_2_ levels one solution may be to add lime to lakes to raise their pH. Unfortunately this often just moves metal pollutants from the water column to sediments (Wällstedt and Borg 2005). Since snails often graze off of sediments, the liming of lakes may actually increase the body burden of metals which may transfer up food chains.

Our results illustrate the difficulty of predicting future effects of rising CO_2_ levels. CO_2_ induced effects add new stressors to already anthropomorphically perturbed system (Gouin et al. 2013; Manciocco et al. 2014) which requires the application of new modes of modelling since adaptions can alter the effects of multiple stressors (Fischer et al. 2013). The result of those added stressors may be non-additive and indirect (Coors and de Meester 2008).

In the future we plan on repeating our LD50 survival experiment with the added treatment of heavy metals. This way snails would be simultaneously challenged by two stressors. We also want to measure cellular and immunological responses. Pulmonate snails have metal-sequestering metallothionein (Dallinger et al. 2001) which provide some tolerance to the toxicants. Perhaps metallothionein is impaired under conditions of hypercapnia.
